# Effects of physical exercise in reducing caregivers burden: a systematic review

**DOI:** 10.3389/fpubh.2025.1474913

**Published:** 2025-02-05

**Authors:** Carla Cardoso, Maria José Lumini, Teresa Martins

**Affiliations:** ^1^Serviço de Oncologia Cirúrgica, Instituto Português de Oncologia do Porto Francisco Gentil, Porto, Portugal; ^2^Escola Superior de Enfermagem do Porto, Porto, Portugal; ^3^CINTESIS&RISE, Porto, Portugal

**Keywords:** family caregivers, exercise program, muscle relaxation, burden, stress, systematic review

## Abstract

**Background and aim:**

Caring for someone can be physically and psychologically demanding, predisposing caregivers to muscle injuries, fatigue, exhaustion, depression, anxiety, and burnout. The literature suggests several approaches to reducing caregiver burden, one of which is physical exercise. The aim of this systematic review was to analyze the effectiveness of exercise-based programs or muscle relaxation in reducing caregiver burden and stress among family caregivers.

**Method:**

A systematic literature review was conducted following the PRISMA guidelines. The search was performed in the Web of Science, Cochrane Library and Scopus databases and through the EBSCOhost aggregator (CINAHL Plus, MEDLINE, and SportDiscus). Studies were selected based on the PICOD acronym.

**Results:**

Eleven randomized controlled trials (RCTs) and two other experimental studies were included. The reviewed programs encompassed aerobics, strengthening, and muscle relaxation exercises, delivered by various professionals in diverse settings, such as caregivers' homes, gyms, and hospital environments. Although the programs varied in type, duration, and structure, the majority of the studies demonstrated positive effects on caregivers' physical and psychological well-being, along with reductions in burden and stress.

**Conclusion:**

The results suggest that physical exercise interventions are effective in reducing caregiver burden and stress, while also enhancing overall well-being. Future strategies should emphasize the importance of raising awareness among caregivers about adopting healthy lifestyles, with a particular focus on regular physical activity, as a means of relaxation and self-care. To maximize the effectiveness of these interventions, incorporating flexible, home-based components and engaging multidisciplinary teams could enhance accessibility, adherence, and impact.

**Systematic review registration:**

https://www.crd.york.ac.uk/prospero/display_record.php?ID=CRD42023446129, PROSPERO 2023 CRD42023446129.

## 1 Introduction

Population aging is a global phenomenon, presenting significant challenges and implications for societies worldwide. The increase in life expectancy and the declining birth rates have led to a higher proportion of older adult individuals in many countries (1). This demographic shift is accompanied by an increase in chronic diseases and disabilities, which often result in a loss of autonomy and a growing need for long-term care and support for activities of daily living (2). The literature highlights the vital role of family caregivers in providing care for older people, especially as the prevalence of age-related conditions such as dementia and physical frailty rises (3). Family caregivers face substantial physical, psychological, and social challenges, which can lead to increased caregiver burden (3).

Like other European countries, Portugal has experienced an increase in the older adult population (4). The most recent data from the 2021 Census shows that the country's aging index is 182 older adults for every 100 young people, with a life expectancy at birth of 83.2 years for women and 78.1 years for men (4, 5). It is estimated that by 2080, Portugal will have the largest number of older adult people in Europe (5). This demographic trend, while reflecting advances in healthcare and living conditions, also presents significant societal challenges, particularly in terms of increased dependency on care and support for activities of daily living. This increased dependency is closely linked to the higher prevalence of chronic diseases and disabilities associated with aging, which often result in loss of autonomy and the need for long-term care (1). Consequently, the last decades of life are marked by physical and/or mental disabilities, making this generation more vulnerable and potentially dependent on daily living activities (6, 7). In Portugal, recent data indicate that in 2023, there were 78,104 older adults living in nursing homes and 76,188 require home care assistance (8). This data shows that there are a substantial number of older adults who depend on family caregivers to meet their basic care needs.

Family caregivers are the greatest collaborators of healthcare professionals, as they allow the dependent person to remain in the community rather than in social institutions. However, family structures have undergone significant changes, including increased participation of women in the workforce, geographic dispersion of family members, and a decline in multigenerational households, all of which have impacted the availability of family members to provide care and shifted traditional caregiving roles (1). This changes in family roles, as well as changes in lifestyles, contribute not only to a reduction in the family's ability to provide care but also to increased caregiver burden (9–11). Caring for someone is a complex process that requires responsibility, exposing the caregiver to physical, psychological, and social stresses, which can lead to significant health impacts (9, 12).

To prevent this trend, it is essential to develop strategies that reduce caregiver burden, enhance self-care, and consequently improve quality of life. The World Health Organization (WHO) has emphasized that self-care measures should be promoted as an individual strategy to achieve high levels of health and well-being in populations (13). An example of this is the UK Department of Health's action plan, which prioritizes self-care as a central element of health services, highlighting that individual involvement leads to better health outcomes by empowering people to care for themselves and take control of their lives (14). Additionally, the non-governmental organization International Self-Care Foundation has developed initiatives in the field of self-care, which are highly relevant for addressing caregiver burden. Conceptually, it identifies seven pillars: health literacy, self-awareness, physical activity, healthy eating, risk prevention or control, hygiene, and the rational and responsible use of products, services, diagnostics, and medications (15).

It is widely agreed that physical activity, the third pillar of self-care, impacts various areas of individual health, such as reducing stress and depressive symptoms, improving overall health even in the presence of chronic diseases, relieving pain, and reducing fatigue, thus enhancing well-being and quality of life, as well as promoting physical and mental health. For family caregivers, regular physical activity allows for an active and healthy life, with moderate-intensity activities reducing psychological stress and caregiver burden, improving health conditions, enhancing physical health, and decreasing the incidence of injuries, pain, or discomfort (16–18). While evidence suggests that interventions promoting physical activity can benefit caregivers by reducing stress, there is insufficient guidance on how to tailor such programs.

Based on the research question: What therapeutic exercises, physical exercise, or muscle relaxation are clinically effective in reducing caregiver burden or stress? A systematic review was conducted with the following objectives: (1) To identify the type of evidence that exists regarding the effectiveness of programs aimed at family caregivers, focusing on physical exercise; and (2) To identify the type and characteristics of these programs in terms of exercises, intensity, and duration.

## 2 Methods

A systematic review was conducted following the Preferred Reporting Items for Systematic Reviews and Meta-analyses (PRISMA) guidelines from the Joanna Briggs Institute (19).

### 2.1 Search strategy

The literature search was conducted using the EBSCOhost aggregator (CINAHL Plus with Full Text, MEDLINE with Full Text, and SportDiscuss) and the Web of Science, Cochrane Library Database and Scopus, from January 1, 2012, to December 19, 2024. The descriptors used correspond to each component of the PICOD strategy and were controlled using the MeSH (Medical Subject Headings) and CINAHL Headings applications to ensure the specificity of the search. The descriptors were further combined with search delimiters using Boolean operators, represented by the terms AND and OR. The following combination was used in each database: [(“caregiver^*^” OR “carer^*^” OR “care giver^*^”) AND (“Exercise Program^*^” OR “Physical Therapeutic exercise” OR “Muscle Relaxation”) AND (“burden” OR “stress”) AND (“Clinical effectiveness” OR “Effectiveness” OR “Systematic Review” OR “Randomized Controlled Trial^*^” OR “RCT” OR “Clinical Effectiveness” OR “Effectiveness” OR “Experimental study”)].

### 2.2 Eligibility criteria

Table 1 summarizes the inclusion criteria for this review, based on the acronym PICOD (participants, intervention, comparison, outcomes, and study design). Participants included all individuals aged 18 and older who were family caregivers of people dependent on self-care. A person dependent on self-care refers to an individual who, due to physical, mental, or emotional limitations, is unable to independently perform activities essential for maintaining their health, safety, and well-being. These activities include, but are not limited to, personal hygiene, eating, dressing, mobility, and basic health care (20). Dependence in self-care can be total or partial, varying according to the severity of the person's condition, and often requires assistance from formal or informal caregivers. Participants (P) should not have any severe acute illness or psychiatric disorders. Studies focusing on children/parents or formal caregivers were excluded, to ensure a more homogeneous sample.

**Table 1:** PICOD search strategy.

**P**	**I**	**C**	**O**	**D**
Family caregivers	Exercise program | Therapeutic exercise muscle relaxation	When applicable, refers to the group of caregivers who did not receive the program	Clinical efficacy in burden or stress	Systematic review/randomized controlled trial (RCT)/experimental study

Regarding the intervention (I), all studies describing exercise programs, sets of therapeutic exercises, or muscle relaxation exercises aimed at family caregivers of people dependent on self-care were considered. Exercise or physical exercise was considered as a structured, planned, and repetitive activity performed to improve or maintain physical fitness, health, or well-being (21). Any type of physical exercise was considered, with or without direct intervention from the professional/researcher involved. As for the outcome (O), the effectiveness (or lack thereof) in reducing levels of burden was considered. The assessment of the effectiveness of the implemented intervention needed to be documented through the description of the instruments used. Regarding the study design, they needed to correspond to systematic literature reviews, randomized controlled trials (RCT), or experimental studies, with other types being excluded. Additionally, studies published in Portuguese, English, or Spanish language, and available in full text, were considered as inclusion criteria.

The protocol for the systematic review is registered in the international database PROSPERO (International prospective register of systematic reviews) with the ID CRD42023446129.

### 2.3 Data extraction

Duplicate studies were removed. Two authors independently reviewed the title and abstract. In the next phase, the full text was also analyzed independently by two authors, and any discrepancies were resolved by a third author. The following information was extracted from the studies: (1) author/year; (2) design/sample; (3) objectives; (4) intervention, frequency, and duration; (5) professional involved; (6) results/efficacy.

### 2.4 Quality assessment

The methodological quality of the studies was assessed using the JBI Critical Appraisal Checklist for Randomized Controlled Trials (22) and the Checklist for Systematic Reviews and Research Syntheses (23). In this phase, two independent reviewers evaluated the methodological quality of the articles, and conflicts were resolved by a third reviewer. Each positively evaluated item was assigned one point, with scores ranging from 0 to 4 points indicating low quality; 5 to 9 indicating medium quality; and 10–13 indicating high methodological quality (Tables 2, 3).

**Table 2:** Methodological quality of the RCT studies.

**Author/Year**	**Loi et al. (33)**	**Çapaci et al. (34)**	**Hives et al. (31)**	**Wang et al. (24)**	**Madruga et al. (28)**	**Montero-Cuadrado et al. (29)**	**Montero-Cuadrado et al. (30)**	**Yilmaz et al. (27)**	**Chan et al. (25)**	**Imanian and Ramezanli (36)**	**Barghbani et al. (35)**
1. Was true randomization used for assignment of participants to treatment groups?	Y	Y	Y	Y	Y	Y	Y	Y	Y	Y	Y
2. Was allocation to treatment groups concealed?	Y	N	N	Y	N	N	N	U	Y	U	N
3. Were treatment groups similar at the baseline?	Y	Y	Y	Y	Y	Y	Y	Y	Y	Y	Y
4. Were participants blind to treatment assignment?	U	N	N	U	N	N	N	U	S	U	N
5. Were those delivering treatment blind to treatment assignment?	N	N	Y	U	U	N	N	U	Y	U	N
6. Were outcomes assessors blind to treatment assignment?	Y	Y	Y	Y	Y	Y	Y	Y	Y	U	Y
7. Were treatment groups treated identically other than the intervention of interest?	Y	N	U	U	U	Y	Y	U	Y	Y	N
8. Was follow up complete and if not, were differences between groups in terms of their follow up adequately described and analyzed?	N	Y	Y	Y	Y	Y	Y	Y	Y	Y	Y
9. Were participants analyzed in the groups to which they were randomized?	Y	Y	Y	Y	Y	Y	Y	Y	Y	Y	Y
10. Were outcomes measured in the same way for treatment groups?	Y	Y	Y	Y	Y	Y	Y	Y	Y	Y	Y
11. Were outcomes measured in a reliable way?	U	Y	Y	Y	Y	Y	Y	Y	Y	Y	Y
12. Was appropriate statistical analysis used?	Y	Y	Y	Y	Y	Y	Y	Y	Y	Y	Y
13. Was the trial design appropriate, and any deviations from the standard RCT design (individual randomization, parallel groups) accounted for in the conduct and analysis of the trial?	Y		Y	Y	Y	Y	Y	Y	Y	Y	Y
Score	9/13	9/13	10/13	10/13	9/13	10/13	10/13	9/13	13/13	9/13	9/13
Y—yes; N—No; U—Unclear											

**Table 3:** Methodological quality of quasi experimental studies.

**Author/year**	**Alonso-Cortés et al. (26)**	**Baykal and Bilgic (32)**
1. Is it clear in the study what is the “cause” and what is the “effect” (i.e., there is no confusion about which variable comes first)?	U	Y
2. Was there a control group?	Y	N
3. Were participants included in any comparisons similar?	Y	U
4. Were the participants included in any comparisons receiving similar treatment/care, other than the exposure or intervention of interest?	U	U
5. Were there multiple measurements of the outcome, both pre and post the intervention/exposure?	Y	Y
6. Were the outcomes of participants included in any comparisons measured in the same way?	Y	Y
7. Were outcomes measured in a reliable way?	Y	Y
8. Was follow up complete and if not, were differences between groups in terms of their follow up adequately described and analyzed?	N	U
9. Was appropriate statistical analysis used?	Y	Y
Score	6/9	5/9
Y—yes; N—No; U—Unclear		

## 3 Results

**Figure 1:**
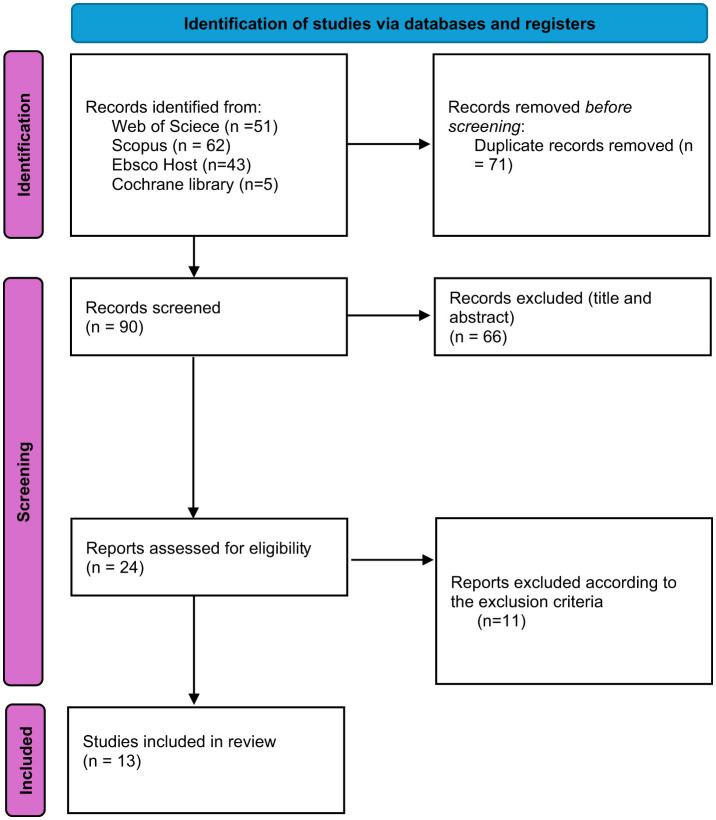
PRISMA flow diagram.

The search yielded 161 studies: 51 from the Web of Science, 62 from Scopus, 5 from Cochrane Library Database and 43 from the EBSCOhost aggregator. After removing duplicate references, 90 studies remained for analysis. Sixty-six articles were excluded after the title and abstract review. Finally, 24 studies were analyzed in full text, of which 11 were excluded based on inclusion and exclusion criteria. In total, 13 articles were included in this systematic review. The process is detailed in Figure 1.

All the studies reported that the majority of the caregivers were female and were spouses or daughters. The mean age of the caregivers' range between 47 (24) and 65 (25).

The education degree of the caregivers ranged from primary school to graduate level. Only some studies mentioned the time spent on caregiving (25, 26), that range according to the health condition of the cared person. The caregiving duration was mentioned in four studies (27–30).

The characteristics of the studies included in this review and the main results are presented in Table 4. The included studies were conducted between 2018 and 2024, with Spain having the highest number of studies (*n* = 4), followed by Turkey (*n* = 3), China (*n* = 2), Iran (*n* = 2), USA (*n* = 1) and Australia (*n* = 1). No study produced in Portugal was found on this topic. The articles were mainly published in the EBSCOhost aggregator, with 11 randomized studies and two quasi-experimental studies. No systematic literature review was found. In the first phase, the following parameters are considered: intervention group, control group, intervention location, professional guiding the program, and whether they actively participate in the intervention by supporting the participants (Table 5).

**Table 4:** Characteristics of the studies, participants, program intervention and results.

**Author, year, country**	**Study design**	**Intervention group (IG)**	**Control group (CG)**	**Program duration/assessment moments**	**Results**
Baykal and Bilgic (32), Turkey	Quasi-experimental study	Progressive muscle relaxation (*n =* 57)	Without control group	3 months, 40 min, 3/week, Total of 36 sessions, Baseline, 3 months	Within-Group analysis: At baseline caregivers displayed elevated levels of stress (*M =* 7.57, SD = 4.13) after intervention the values decreased significantly (*M =* 5.08, SD = 5.69; *p =* 0.000). Caregiver burden was significantly high at baseline (*M =* 61.80, SD = 15.58) then after the intervention (*M =* 29.85, SD = 5.10; *p =* 0.000).
Loi et al. (33), Australia	RCT with 3:3:1 allocation	Individualized program based on the Otago-Plus Exercise Program (*n =* 34)	Social control (the same program of IG without the exercise program) (*n =* 42) Usual care (*n =* 15)	6 months, 30 min, 5 days per week, Baseline, 6 months	Between Groups analysis: IG vs. Social Control comparison showed no significant difference (regression coefficient: −1.64, 95% CI: −6.20 to 2.92, *p =* 0.48). IG vs. Usual Care Group comparison also showed no significant difference (regression coefficient: 4.56, 95% CI: −1.12 to 10.24, *p =* 0.11). Within-Group analysis: IG decreased from 41.3 to 39.9 (change of −1.4 points). In Social Control group increased from 37.5 to 38.1 (change of +0.6 points). In Usual Care Control group decreased from 35.8 to 29.9 (change of −5.9 points). The results of intra-subject analysis show no significant differences between groups over time.
Barghbani et al. (35), Iran	RCT	Benson relaxation program (*n =* 57)	No intervention (*n =* 56)	1 month, 20 min, 2/day, Total of 60 sessions, Pre, post program	Between Groups analysis: All dimensions of caregiver burden were significantly lower after the intervention. The total score decreased to 57.21 ± 14.67 in the intervention group, while it slightly increased to 75.47 ± 13.95 in the control group (*p =* 0.000). Within-Group analysis: The results showed a significant reduction in total caregiver burden within the intervention group before and after the intervention (*p =* 0.000). In the CG, no significant change was observed in the total burden (*p =* 0.519). The most pronounced reduction occurred in emotional burden.
Montero- Cuadrado et al. (29, 30), Spain	RCT	Therapeutic exercise program combined with conventional theoretical sessions (*n =* 32)	Conventional theoretical sessions (*n =* 30)	12 weeks, 90 min, 3/week, Total of 36 sessions, Pre, post program	Between Groups analysis: After the intervention, the IG showed significantly greater improvements compared to the CG. The total subjective caregiver burden was 10.32 points lower in the IG than in the CG (*p* < 0.001; effect size = −2.38). Within-Group analysis: The total subjective caregiver burden decreased significantly in IG after the intervention, from 57.06 ± 13.45 to 48.46 ± 11.19 (*p* < 0.001). In the CG, the total subjective caregiver burden showed a slight, non-significant increase (from 54.93 ± 15.40 to 56.67 ± 15.13, *p* > 0.05).
Imanian and Ramezanli, (36), Iran	RCT	Benson relaxation program (*n =* 24)	No intervention (*n =* 24)	1 month, 15 min, 2/day, Total of 60 sessions, pre, post program	Between groups analysis: after the intervention: the caregiver burden was significantly lower in the intervention group (14.46 ± 10.91) than in the control group (34.96 ± 15.99), with *p* < 0.001. Within-group analysis: there was a significant reduction in caregiver burden after the intervention, from 38.33 ± 16.94 to 14.46 ± 10.91 (*p* = 0.001). While in CG there was no significant change in caregiver burden before and after the study (35.75 ± 19.33 vs. 34.96 ± 15.99; *p* = 0.84).
Çapaci et al. (34), Turkey	RCT	Progressive muscle relaxation program (*n =* 10)	No intervention (*n =* 10)	8 weeks, 30 min, 4/ week, Total of 32 sessions, pre, post program	Between groups analysis: after the intervention, the burden scores of the IG were significantly lower than that of the CG (*p =* 0.011). Within-group analysis: In the IG there was no statistically significant difference between the pre and post-intervention mean ZBI scores (*p =* 0.214). In the CG, a statistically significant increase in the mean ZBI score was observed between the pre- and post-intervention periods (*p =* 0.002), indicating a worsening of caregiver burden in the absence of the intervention. Interaction between group and time: there was no overall significant difference in ZBI scores between the IG and CG across time (*p =* 0.110). However, a significant change in ZBI scores was observed over time (*F =* 8.661, *p =* 0.009). Importantly, the group × time interaction was statistically significant (*F =* 18.365, *p* < 0.001), indicating that the changes in ZBI scores over time differed significantly between the IG and CG.
Hives et al. (31), USA	RCT	Individual aerobic exercise program (*n =* 34)	No intervention (*n =* 34)	24 weeks, 20–30 min, 3/week, minimum of 27 sessions in total, pre, post program	Between groups analysis: a significant treatment effect was observed in the aerobic exercise group when compared to the CG, with an unstandardized treatment effect of −4.60 (95% CI: −8.82, −0.38, *p* < 0.05). Within-group analysis: in the aerobic exercise group, caregiver burden significantly decreased over time, with a change of −6.59 (95% CI: −9.62, −3.55, *p* < 0.05). The CG did not show significant changes in caregiver burden, with a change of −1.99 (95% CI: −4.92, 0.95).
Wang et al. (24), China	RCT	Progressive muscle relaxation program (*n =* 55)	Booklet with rehabilitation advice and verbal counseling (*n =* 55)	12 months, 90 min, 2/month, total of 24 sessions, M0, M3, M6, M12 months	Between Groups analysis: In M0 and M3 there was no significant difference in caregiver burden between the IG and CG. In M6 and M12, the IG exhibited a significant reduction in caregiver burden compared to the CG, with a *p*-value at M6 (*p =* 0,046) and a value (*p =* 0.009) at M12. Within-group analysis: IG:M0 to M3 increase; M3 to M6 increase; M6 to M12 decrease. CG: M0 to M3 increase; M3 to M6 increase; M6 to M12 increase.
Madruga et al. (28), Spain	RCT	Aerobic, strength, and relaxation exercise program (*n =* 25)	No intervention (*n =* 23)	9 months, 60 min, 2/week, total of 72 sessions, baseline, after program	Between groups analysis: after the 9-month intervention, a significant reduction in subjective burden was observed in the IG (47.80 ± 11.04) compared to the CG (56.13 ± 14.30) (*p* < 0.01). The effect size (Cohen's *d*) was −0.572, indicating a medium to large impact of the intervention on reducing burden. Within-group analysis: In IG subjective burden significantly decreased from baseline (55.7 ± 12.4) to the end of the intervention (47.8 ± 11.0) (*p* = 0.001). In contrast, there was no significant change in the CG, with baseline scores of 55.7 ± 14.7 and post-intervention scores of 56.1 ± 14.3.
Chan et al. (25), China	RCT	12-step sitting Tai Chi aerobic exercise program (*n =* 69)	No intervention (*n =* 68)	24 weeks, 60 min, 1/week for the first 4 weeks and then every 15 days. Baseline, 6, 12, and 24 weeks	Between groups analysis: no significant differences in caregiver burden between Tai Chi and social contact groups at both week 6 (interaction effect: −0.21, SE = 2.16, *p* > 0.05); Week 12 (interaction effect: 0.15, SE = 1.90, *p* > 0.05) and Week 24 (interaction effect: 1.01, SE = 0.87, *p* > 0.05). Within-group analysis: in IG there was an initial reduction (baseline to week 6), followed by a progressive increase until week 24. This suggests that the initial positive impact may have been transient. In CG an initial reduction occurred, but with minor fluctuations and a slight stabilization at week 24.
Alonso-Cortés et al. (26), Spain	Pre-experimental study the groups were not randomized	Program with theoretical sessions and practical relaxation workshops (*n =* 22)	Theoretical sessions only (*n =* 14)	4 months, 60–90 min,1/week. Total of 14 sessions. Pre, post program; pre training, follow up 36 weeks; post training, follow up	Between groups analysis: at the post-training, the IG demonstrated significantly lower burden scores (*p =* 0.017). The follow-up assessment, the IG continuing to exhibit a significantly lower global burden (*p =* 0.016). Specifically, the mean global burden scores for the IG decreased from 50.09 ± 11.36 post-training to 49.23 ± 11.53 at follow-up, while the CG maintained higher scores of 61.43 ± 13.74 and 60.57 ± 12.17, respectively. Within-group analysis: In the IG, a decrease in burden was observed between the pre- and post-program periods (*p* < 0.001) and between the pre-program and follow-up periods (*p* < 0.001), but no statistically significant differences were found between the post-training and follow-up periods. In the CG, no statistically significant differences were found in the variation of burden.
Yilmaz et al. (27), Turkey	RCT	Progressive muscle relaxation program (*n =* 23)	No intervention (*n =* 21)	8 weeks, 28 min, 3/week. Total of 24 sessions. Baseline, pos program	Between groups analysis: after the intervention, the difference in mean scores between the intervention (42.89 ± 10.86) and control (41.33 ± 15.24) groups was not statistically significant. Within-group analysis: the IG demonstrated a statistically significant reduction in caregiver burden scores from the preliminary evaluation (49.66 ± 12.42) to the final evaluation (42.89 ± 10.86) (*p =* 0.001), while the CG did not show a significant change (*p =* 0.092).

**Table 5:** Characteristics of the intervention.

**Target population**	**Location of application**	**Guiding professional**	**Direct presence of the professional**
Dementia/Alzheimer's (25, 26, 28, 31, 32)	Home (25, 27, 28, 33–36)	Nurses (27, 34–36)	Programs with the presence of professionals (25, 26, 28–30, 32)
Dependent older adults (29, 30, 33, 34)	Caregiver associations (26, 29, 30)	physical therapists (26, 29, 30)	Programs without the presence of professionals (27, 31, 33–36)
Post-stroke people (24, 27)	Gym/home and hospital/home (24, 31)	Personal trainer (28, 31)	Not specify (24)
Chronic patients (35, 36)	Not specify (32)	Tai Chi instructor (25)	
		Not specify (24, 32)	

Next, each of the programs established in the studies was analyzed concerning its duration, total number of sessions, duration of each session, interval between sessions, and the type/description of exercises performed, as well as the type of exercise developed in each program (Table 6).

**Table 6:** Program characteristics.

**Program type**	**Program duration**	**Total number of sessions**	**Frequency**	**Session duration**
Combined physical exercise	4–9 months	8–72	2-3/week	60–90 min
Aerobic exercise	Up to 24 weeks	8–27	1-3/week	20–60 min
Muscle relaxation	1–12 months	24–60	1/day−2/month	15–90 min

Two publications are related to the same study (29, 30) The majority of studies were directed at caregivers of people with Alzheimer's or other types of dementia (25, 26, 28, 31, 32); four studies were directed at caregivers of dependent older adults (29, 30, 33, 34); two studies were related with caregivers of people who suffered a stroke (24, 27); and two studies were directed at caregivers of chronic patients, with cancer (35) or undergoing hemodialysis (36) (Table 5).

The samples were heterogeneous regarding the number of participants, ranging from 20 to 137 participants. Concerning the implemented intervention, following the authors' classifications, was grouped into three categories: combined physical exercise programs (28–30, 33), consisting of aerobic, strength, and relaxation activities; aerobic exercise programs (25, 31); and muscle relaxation programs (24, 26, 27, 32, 34–36). The number of participants in the intervention and control groups was similar, except one study with an allocation 3:3:1 (33). Only one study doesn't have a control group (32) have an equivalent group (26). All control groups of the analyzed studies did not have interventions using physical activity.

The intervention took place mainly at home (25, 27, 28, 33–36), followed by studies where the intervention was developed in caregiver associations (26, 29, 30, 32), and studies conducted in a gym/home (31)and hospital/home (24).

The professionals who guided the programs for family caregivers were physical therapists (26, 29, 30, 33) nurses (27, 34–36); personal trainers (28, 31); and a Tai Chi instructor (25). In two studies, the professional guiding the program was not identified (24, 32) (Table 5).

In six of the analyzed programs, the intervention was conducted after the initial training without the direct presence of the professional (27, 31, 33–36). These correspond to four muscle relaxation programs (27, 34–36), one aerobic exercise program (31), and one a combined program (33) where support materials such as training plans, audio/CD files, and pamphlets were provided (Table 5).

Except for one study that did not have a control group, all others stated that, at baseline, the intervention group was similar to the control group, with no statistically significant differences in clinical variables analyzed. In two studies the program was aimed at caregivers and people cared for (25, 33).

### 3.1 Combined exercise programs

Considering the studies on composite physical exercise programs that include aerobic, strength, and relaxation components (28–30, 33), it is observed that these differ in terms of their duration, total number of sessions, and intervals between sessions. However, it is noted that this type of program tends to have an extended duration, ranging from 4 to 12 months, with a total of sessions ranging from 8 to 72.

Exercise sessions were held two to five times per week. The programs consisted of a set of similar activities, including a warm-up period, aerobic exercises, followed by strength exercises, and finally relaxation exercises. Participants' heart rates were monitored, and the intensity of the exercises was adjusted accordingly. One study combined the physical activity program with conventional theoretical training for caregivers, addressing topics such as caregiving, available social resources, and caregiver self-care education (29, 30).

### 3.2 Aerobic exercise programs

The two aerobic exercise programs analyzed had a total duration of 24 weeks (25, 31), with the number of sessions varying between 8 (25) and 27 (31). Each session lasted from 20 to 60 min, with the interval between sessions ranging from one to three times per week. The exercises in the two programs were very different: one program included activities such as walking, running, cycling, or swimming, with progression in intensity and duration (31), while the other involved a Tai Chi program where participants performed the exercises seated (25). The Tai Chi program was considered aerobic exercise according to the authors' classification and its results didn't show significant differences between groups or within groups (25).

### 3.3 Muscle relaxation programs

The muscle relaxation programs analyzed (24, 26, 27, 32, 34–36)also showed significant variability. The total duration of the programs ranged from 1 to 12 months, with a total number of sessions ranging from 24 to 60. Sessions lasted between 15 and 90 min, and the interval between sessions varied from twice a day to twice a month. Applying the JBI Critical Appraisal Checklist for Randomized Controlled Trials showed that seven studies have medium methodological quality and five have high methodological quality (Tables 2, 3).

## 4 Discussion

The programs analyzed differ in their type; however, all the studies converge on a common goal, which is to evaluate the effectiveness of interventions on burden and stress of informal caregivers of people dependent on self-care.

The studies analyzed were mainly conducted in Spain (26, 28–30), which may be related to the greater recognition of physical activity in that country as a protective factor for mental health and caregiver burden. It is noteworthy that regular physical activity positively contributes to all age groups, promoting not only physical and mental health but also maintaining weight, enhancing well-being, and reducing anxiety and depression (13, 17, 20, 21). Throughout the life cycle, engaging in physical activity according to WHO guidelines acts as a protective factor against various chronic diseases and as an adjunct to their treatment (21).

In six of the programs, caregivers performed the suggested interventions without the direct presence of a professional, four of them had results clearly positive (31, 34–36). Of these, three studies, reported to muscle relaxation programs (34–36). This is significant because it can serve as a reference for implementing more economically accessible programs with the potential to be implemented on a large scale. It is emphasized that developing interventions on scientifically validated web platforms can represent a new strategy of efficient support for caregivers, considering their limited leisure time and the difficulty of leaving home due to caregiving responsibilities (37). However, home-based programs without the supervision of a healthcare professional may not be as effective since real-time monitoring and correction are not performed. Additionally, they may not be as attractive since participants do not experience group motivation (38). Nevertheless, they represent a possibility of reaching a larger target audience since they require fewer resources and professionals involved. Puterman et al. (39) argue that, to overcome these issues, supervision through communication devices such as phone calls and messages can be used to monitor and motivate family caregivers to engage in the proposed program.

Although muscle relaxation programs may not traditionally be classified as physical exercise, the systematic contraction and relaxation of muscle groups involved in these methods induce physical activity and contribute to overall well-being (32). Two studies employing Benson's relaxation technique demonstrated significant reductions in caregiver burden (35, 36). Among the studies utilizing progressive muscle relaxation programs, only one did not show significant inter-group differences; however, it did reveal significant intra-group differences (27).

The duration of the programs ranged from 1 to 12 months, with frequencies of 2–5 times per week. The variability in results suggests that both duration and frequency may influence the effectiveness of the interventions, highlighting the importance of considering the dose-response relationship when prescribing programs for caregivers. Additionally, combined exercise programs that include both physical and theoretical components (26, 29, 30) may offer greater benefits. The Otago exercises did not show significant results either in the between-group analysis or in the within-subject analysis (33). However, in this study, the caregivers had a high burden at the outset (i.e., a score above 5 on the Geriatric Depression Scale), which may have influenced the final results (33).

The programs analyzed were primarily developed by professionals other than nurses. More systemic approaches involve different professionals, notably those more connected to sports. However, nurses have shown they are prepared to face these challenges, as demonstrated in the studies (27, 34–36). Nurses play an important role in monitoring and improving caregivers' physical activity, contributing not only to their functional independence but also to their quality of life (40). A partnership between professionals and community resources facilitates the development and implementation of activities that promote physical activity among family caregivers (40, 41).

It's important to note that while some studies reported significant within-group improvements, between-group analyses did not always show significant differences. This indicates that individual responses to interventions can vary, and the presence of a control group is crucial for accurately assessing effectiveness.

Longer and more intensive intervention programs tend to be more effective in reducing caregiver burden. For example, a 9-month program combining aerobic, strength, and relaxation exercises, totaling 72 sessions, resulted in a significant reduction in subjective caregiver burden in the intervention group compared to the control group, with an effect size of −0.572, indicating a medium to large impact on burden reduction (28). In contrast, shorter or less intensive programs have shown less consistent results. Additionally, the maintenance of positive effects appears to be related to the continuity of interventions. Wang et al. observed significant reductions in caregiver burden at 6 and 12 months compared to the control group, but after the program, burden levels tended to increase (24).

The fact that the physical exercise programs were designed for both the caregivers and the cared-for individuals (25, 33)may also haven't a significant, as this activity was not exclusively dedicated to the caregivers' time.

Regarding the methodological quality of the studies, there were variations in allocation concealment and blinding of participants, treatment providers, and outcome assessors, which impacts the risk of bias. The study by Chan et al. (25), stood out as the only one meeting all the criteria (13/13), indicating high methodological quality, while other studies scored lower (9/13), with critical issues such as allocation concealment (not met by several) and inconsistencies in identical treatment between groups. Studies with lower scores may be less reliable due to a higher risk of bias, requiring caution in interpreting their results. Quasi-experimental studies show significant vulnerabilities in their methodological quality.

As limitations of this review, it is noted that only studies in Portuguese, English, and Spanish language available in full text were included. This language restriction may have led to the exclusion of relevant studies published in other languages, potentially introducing a selection bias. Additionally, the defined time frame and selected databases may have limited the research conducted. The established exclusion criteria could have restricted the inclusion of potentially relevant publications in the literature review. Furthermore, the search terms adopted, although carefully selected, may not have encompassed all variations or expressions associated with the theme, which may have resulted in omissions or divergent results.

## 5 Conclusion

The aging population presents multiple challenges in health and social sectors. This process is accompanied by situations of dependency or disability, primarily caused by chronic non-communicable diseases and their complications. Consequently, the number of family caregivers in society is progressively increasing, exposing them to continuous physical, psychological, and social strain, which inevitably leads to caregiver burnout. The findings suggest that physical exercise programs can positively impact caregivers' physical and psychological well-being, reducing stress and burden. Notably, combined exercise programs and muscle relaxation techniques showed promising results in alleviating caregiver burden. Additionally, interventions that allowed caregivers to perform exercises independently at home, without direct professional supervision, demonstrated potential for broader implementation due to their cost-effectiveness and accessibility. However, the review also highlights the need for more long-term studies to assess the sustained effects of these interventions. Furthermore, the limited involvement of nurses in developing and guiding these programs suggests an opportunity for greater interdisciplinary collaboration in supporting informal caregivers.

Developing strategies to prevent or mitigate this trend is essential for enhancing caregivers' self-care, self-esteem, and well-being. Therefore, it is necessary to raise awareness among caregivers about adopting healthy lifestyles, including regular, enjoyable physical activity as a strategy for relaxation and self-care.

In conclusion, physical exercise interventions appear beneficial in reducing the burden and enhancing the well-being of informal caregivers. Future programs should consider incorporating flexible, home-based components to increase accessibility and adherence, and involve a multidisciplinary team to address the diverse needs of caregivers effectively.

## Data Availability

The original contributions presented in the study are included in the article/supplementary material, further inquiries can be directed to the corresponding author.
